# Discordance Between Radiological and Pathological Responses to Pembrolizumab in Mismatch Repair-Deficient Metastatic Colorectal Cancer: Implications for Precision Oncology

**DOI:** 10.3390/cancers17132233

**Published:** 2025-07-03

**Authors:** Yoshifumi Shimada, Mae Nakano, Akio Matsumoto, Hikaru Ozeki, Kaoru Abe, Yosuke Tajima, Daisuke Yamai, Hitoshi Nogami, Masato Nakano, Tatsuo Tani, Mikako Kawahara, Atsushi Nishimura, Yuka Kobayashi, Yuta Bamba, Susumu Suzuki, Hidehito Oyanagi, Taku Ohashi, Hitoshi Kameyama, Akira Iwaya, Hiroshi Ichikawa, Jun Sakata, Toshifumi Wakai

**Affiliations:** 1 Division of Digestive and General Surgery, Niigata University Graduate School of Medical and Dental Sciences, Niigata 951-8510, Japanmasatona@med.niigata-u.ac.jp (M.N.); y-bamba@med.niigata-u.ac.jp (Y.B.); hichikawa-nii@med.niigata-u.ac.jp (H.I.); jsakata2@med.niigata-u.ac.jp (J.S.); wakait@med.niigata-u.ac.jp (T.W.); 2 Department of Genome Medicine, Niigata University Medical and Dental Hospital, Niigata 951-8520, Japan; 3 Department of Gastrointestinal Surgery, Niigata Cancer Center Hospital, Niigata 951-8133, Japan; hnogami@niigata-cc.jp; 4 Department of Surgery, Nagaoka Red Cross Hospital, Nagaoka 940-2085, Japan; 5 Department of Surgery, Nagaoka Chuo General Hospital, Nagaoka 940-8653, Japan; 6 Department of Oncology, Nagaoka Chuo General Hospital, Nagaoka 940-8653, Japan; 7 Department of Surgery, Niigata Prefectural Central Hospital, Joetsu 943-0192, Japan; 8 Department of Surgery, Niigata Prefectural Shibata Hospital, Shibata 957-8588, Japan; 9 Department of Digestive Surgery, Niigata City General Hospital, Niigata 950-1197, Japaniwa@hosp.niigata.niigata.jp (A.I.)

**Keywords:** pembrolizumab, mismatch repair deficient, microsatellite instability-high, colorectal cancer, discordance, radiological response, pathological response, clinical complete response, pathological complete response

## Abstract

**Background/Objectives**: Pembrolizumab demonstrates a high response rate in patients with mismatch repair-deficient (dMMR) metastatic colorectal cancer (mCRC), with responses often sustained even after treatment cessation. However, the pathological complete response (pCR) rate in clinically responding cases remains unknown. This study aimed to evaluate the relationship between the radiological and pathological responses in patients with dMMR mCRC who responded to pembrolizumab. **Methods**: This retrospective study included 27 patients with dMMR mCRC treated with pembrolizumab. The radiological response was assessed using RECIST version 1.1 criteria. The pathological response was evaluated in patients who underwent metastasectomy, with pCR defined as the absence of residual cancer cells. **Results**: The median progression-free survival (PFS) of the cohort was 19 months. Among the 27 patients, 3 achieved clinical complete response (cCR), and 10 had clinical partial response (cPR), resulting in a response rate of 48%. All three patients with cCR maintained their responses without metastasectomy (2-year PFS: 100%). Among patients with cPR, eight maintained their responses, while two experienced progression (2-year PFS: 75%). Five patients with cPR underwent curative-intent metastasectomy, and four of them (80%) achieved pCR. Thus, at least 40% of patients with cPR in this cohort had no residual cancer histologically. **Conclusions**: Our findings demonstrate the significant discordance between the radiological and pathological responses to pembrolizumab in dMMR mCRC patients, with 80% of those with cPR achieving pCR upon metastasectomy. These observations highlight the need for improved response assessment methods for precision immunotherapy.

## Simple Summary

Pembrolizumab has established its place as a tissue-agnostic treatment for patients with unresectable metastatic mismatch repair-deficient (dMMR) solid tumors, offering higher response rates and more durable therapeutic effects compared to those for chemotherapy in dMMR metastatic colorectal cancer (mCRC). While its clinical efficacy is well-documented, there is a critical knowledge gap regarding the correlation between the radiological and pathological responses in these patients. Hence, we investigated the relationship between the radiological and pathological responses in 27 patients with dMMR mCRC treated with pembrolizumab, with a particular focus on patients who underwent metastasectomy following pembrolizumab treatment. Our key finding is that four out of five (80%) patients with clinical partial response who underwent metastasectomy achieved pathological complete response, indicating a significant discordance between the radiological assessment and actual tumor response. This discordance between the radiological and pathological responses suggests that some patients with radiological partial response may have experienced the complete eradication of viable tumor cells.

## 1. Introduction

Despite the established efficacy of pembrolizumab in mismatch repair-deficient (dMMR) metastatic colorectal cancer (mCRC), emerging evidence raises several issues regarding immune checkpoint inhibitor (ICI) therapy in this setting. The microsatellite instability (MSI) status may not remain stable over time, with reports of microsatellite instability-high (MSI-H)-to-microsatellite-stable (MSS) conversion during tumor evolution and treatment pressure, potentially affecting the long-term treatment efficacy [1]. Additionally, cancer cell metabolism undergoes significant changes during immunotherapy, which may influence the tumor microenvironment and immune cell functions [2]. These metabolic reprogramming events can potentially affect the treatment response independent of the MSI status [3]. Furthermore, accumulating evidence suggests that the current biomarkers, including the MSI status and tumor mutational burden, may not be truly predictive for ICI efficacy in all patients, as a subset of metastatic MSI-H patients demonstrate primary resistance while some MSS patients occasionally respond to immunotherapy [4]. These observations highlight the complexity of predicting the immunotherapy response and underscore the need for more comprehensive biomarker strategies beyond traditional MSI testing.

Pembrolizumab, an anticancer immunotherapy agent, has been applied as a tissue-agnostic treatment for patients with unresectable metastatic dMMR solid tumors [5,6,7]. Pembrolizumab was approved by the Food and Drug Administration in May 2017 for the treatment of unresectable or MSI-H or dMMR solid tumors that have progressed following prior treatment. In mCRC, pembrolizumab is recognized as standard care for systemic therapy in both first-line [8,9] and second-line [10,11] treatments. The first-line treatment of pembrolizumab for dMMR mCRC has two features compared to chemotherapy: first, pembrolizumab showed a higher response rate than that of chemotherapy, and second, the therapeutic effect of pembrolizumab lasted longer than that of chemotherapy [8,9]. However, these pivotal studies provide no detailed data regarding the pathological response after treatment with pembrolizumab.

A dissociation between the radiological and pathological responses has been noted after pembrolizumab treatment. Ludford et al. reported the pathological responses of 14 patients with dMMR mCRC who underwent metastasectomy after treatment with ICIs, including five patients treated specifically with pembrolizumab [12]. Of these, four had persistent tumors visible on imaging, but histopathological analysis revealed no residual cancer [12]. This finding suggests that even when imaging indicates the presence of cancer following pembrolizumab treatment, histopathological examination can demonstrate the complete absence of viable tumor cells.

This issue has also been observed in neoadjuvant immunotherapy settings [13,14,15,16]. Recent studies on neoadjuvant ICIs in CRC have shown that radiological assessment often underestimates pathological response rates. For instance, Pei et al. reported that seven out of eight (88%) patients with partial response or stable disease on imaging achieved pathological complete response (pCR) following neoadjuvant anti-PD-1 therapy [13], suggesting that the discordance between radiological and pathological responses represents a fundamental limitation of the current assessment methods in the immunotherapy era.

Conventional response assessment using RECIST 1.1 criteria, however, may not adequately capture the complex response patterns characteristic of immunotherapy. Unlike chemotherapy, which typically induces rapid tumor shrinkage, immunotherapy can produce delayed responses, mixed responses, and even initial tumor enlargement (pseudoprogression) followed by subsequent response [17]. These atypical response patterns may lead to the underestimation of the treatment efficacy when assessed using conventional RECIST 1.1 criteria. However, RECIST 1.1 remains the standard assessment method in routine care, highlighting the need to understand its limitations in immunotherapy settings.

Several ICIs beyond pembrolizumab have shown efficacy in dMMR mCRC. Nivolumab monotherapy demonstrated an objective response rate of 31% in CheckMate 142 [18], while the combination of nivolumab plus ipilimumab achieved a higher response rate of 55% [19]. Dostarlimab demonstrated remarkable efficacy in locally advanced dMMR rectal cancer, achieving 100% cCR [20]. Emerging therapies under clinical investigation include novel combination strategies with anti-angiogenic agents [21]. Multiple clinical trials continue to investigate pembrolizumab in various settings for dMMR mCRC, including perioperative approaches and novel combination strategies [22]. These investigations may influence future response assessment approaches and treatment strategies. While pembrolizumab has the most extensive clinical data on ICIs used for dMMR mCRC patients, the radiological–pathological discordance we investigate may represent a phenomenon relevant to all immunotherapeutic approaches in this setting.

While pembrolizumab is effective in dMMR mCRC [8,9,10,11] and its therapeutic effects often persist after cessation [5], limited data exist on the pathological responses of patients who respond clinically. Specifically, the pCR rate among clinically responding patients is unknown. This study aimed to evaluate the discordance between the radiological response assessed by RECIST 1.1 criteria and the pathological response in patients with dMMR mCRC treated with pembrolizumab, thereby investigating the limitations of conventional radiological assessment in immunotherapy.

## 2. Materials and Methods

### 2.1. Patients

This retrospective study was performed in accordance with the Helsinki Declaration, and it was approved by the institutional review boards of all participating hospitals (approval number: 2018-0137). Due to its retrospective observational design, the Ethics Committee waived the requirement for written informed consent. Instead, study details were disclosed on the Niigata University website, allowing patients the opportunity to opt out of participation. This study included 27 patients with dMMR mCRC who were treated with pembrolizumab at Niigata University Medical and Dental Hospital and its affiliated hospitals between January 2019 and December 2023. Among these, two cases have been previously reported: one case of metastatic colon cancer achieving complete pathological response [23] and one case of conversion therapy for the peritoneal metastasis of rectal cancer in a patient with Lynch syndrome [24].

### 2.2. MMR Status Assessment

The MMR status was evaluated using MSI testing. The MSI testing was performed on tumoral DNA using polymerase chain reaction (PCR) with five markers (BAT25, BAT26, NR-21, NR24, and MONO-27). MSI-H was defined as the presence of instability at two or more loci.

### 2.3. Radiological Response and Pathological Response

The radiological response was primarily evaluated using computed tomography (CT) scans following the criteria of RECIST version 1.1. The CT scans were performed every three months to assess the disease progression. All imaging was performed with standardized protocols using multidetector CT scanners with a slice thickness of 5 mm for the chest, abdomen, and pelvis, which is in accordance with the RECIST 1.1 criteria for measurable lesions. All imaging studies were reviewed by a dedicated gastrointestinal oncology specialist with over 10 years of experience in RECIST evaluation, who was blinded to the pathological outcomes at the time of the radiological assessment. Due to the retrospective study design, independent dual review was not performed. The treatment response was determined solely based on RECIST 1.1 criteria, without consideration of the biochemical or clinical parameters. The pathological response was determined by assessing the presence of pCR after metastasectomy. pCR was defined as the complete absence of residual cancer cells upon histopathological examination.

### 2.4. Statistical Analysis

All statistical analyses were conducted using IBM SPSS Statistics version 28 (IBM Japan, Inc., Tokyo, Japan). The progression-free survival (PFS) rates were estimated using the Kaplan–Meier method, and the differences between the subgroups were evaluated with the log-rank test. A *p*-value < 0.05 was considered statistically significant.

## 3. Results

### 3.1. Clinicopathologic Description

A total of 27 patients with dMMR mCRC were included in this study (Table 1). The median age at the time of treatment was 69 years. MSI testing was performed on samples for all 27 patients, which showed that all were MSI-H. Of the patients, 14 (52%) had synchronous metastasis, while 13 (48%) had metachronous metastasis. Nineteen patients (70%) had one metastatic organ, and eight patients (30%) had two or more metastatic organs. Pembrolizumab was administered as the first-line treatment to 12 patients (44%) and as the second-line treatment to 15 patients (56%). Five patients (19%) underwent metastasectomy with curative intent following pembrolizumab treatment.

### 3.2. Objective Response and Overall Response

Among the 27 patients, 3 achieved clinical partial response (cCR) and 10 had clinical partial response (cPR), resulting in an objective response rate of 48% (Table 2).

### 3.3. Progression-Free Survival According to Radiological Response and pCR in Patients with cPR

**Figure 1:**
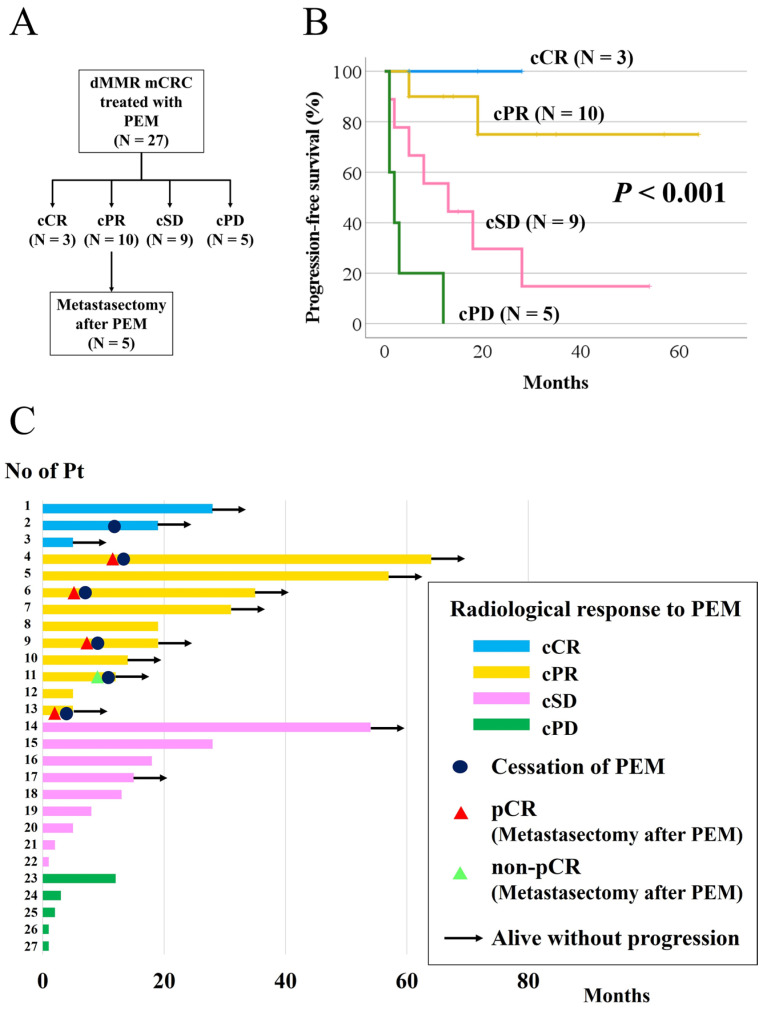
Radiological response and pathological response to pembrolizumab. (**A**) Flowchart of patients included in this study: The flowchart consists of a diagram showing the radiological responses to pembrolizumab and metastasectomy in this cohort. (**B**) Progression-free survival according to radiological response to pembrolizumab: The Progression-free survival rates were categorized by the radiological response to pembrolizumab treatment. (**C**) Swimmers’ plot of progression-free survival according to radiological response to pembrolizumab: (1) Sample sizes per group were as follows: cCR (*n* = 3), cPR (*n* = 10), cSD (*n* = 9), and cPD (*n* = 5). (2) This plot shows the individual Progression-free survival times and responses in patients categorized by the radiological response to pembrolizumab.

**Figure 2:**
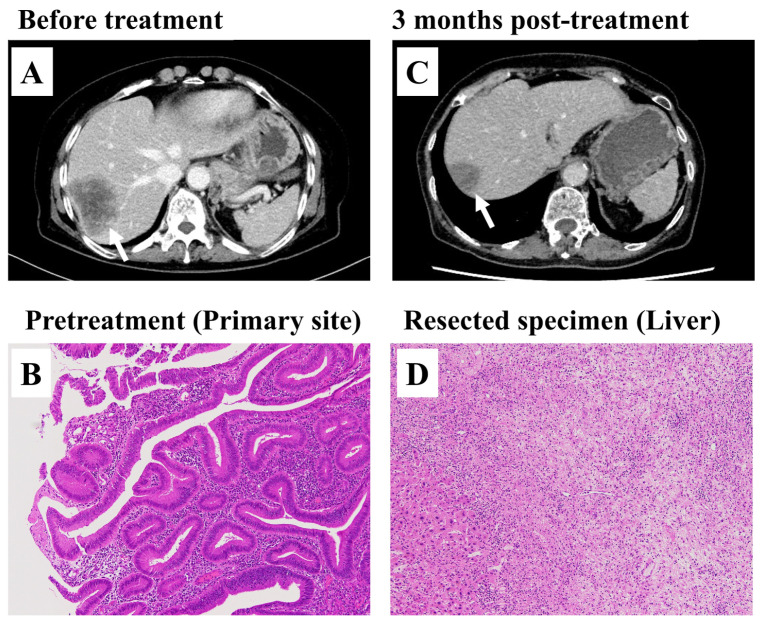
Representative case of metastasectomy after pembrolizumab treatment. (**A**) Radiological imaging of liver metastasis prior to pembrolizumab treatment, highlighting areas of concern (arrow). (**B**) Histopathological image of the primary specimen before treatment. (**C**) Post-treatment radiological imaging of liver metastasis, diagnosed as clinical partial response with a reduction in tumor size (arrow). (**D**) Histopathological image of the resected liver specimen after pembrolizumab treatment, showing no viable cancer cells.

The median PFS for the cohort was 19 months. Five patients with cPR underwent curative-intent metastasectomy (Figure 1A). All three patients with cCR maintained their responses without metastasectomy (2-year PFS: 100%) (Figure 1B). Six patients discontinued pembrolizumab treatment and are alive without progression (Figure 1C). Among the patients with cPR, eight maintained their responses, while two experienced progression (2-year PFS: 75%) (Figure 1B,C). Of the five patients with cPR who underwent curative-intent metastasectomy, four (80%) achieved pathological complete response (pCR) (Figure 2, Table 3 and Table 4). Therefore, at least 40% of patients with cPR had no residual cancer histologically.

This case represents a female patient in her 70s with dMMR metastatic colorectal cancer who achieved pathological complete response following pembrolizumab treatment. The patient had cecal cancer with synchronous multiple liver metastases and underwent ileocecal resection followed by pembrolizumab therapy (three cycles at 6-week intervals). After pembrolizumab treatment, radiological imaging showed partial response, leading to curative-intent metastasectomy.

**Table 3:** Characteristics of patients undergoing metastasectomy after pembrolizumab treatment.

Characteristic		*n* (%)
Radiologic response	cPR	5 (100)
Pathological response	pCR	4 (80)
	non-PCR	1 (20)

*cPR*: clinical partial response; *pCR*: pathological complete response; *non-pCR*: non-pathological complete response.

**Table 4:** The clinicopathological characteristics of five patients who underwent metastasectomy after pembrolizumab treatment.

No. of Pt	Age	Sex	Primary Tumor Location	*KRAS* Status	*BRAF* Status	Site of Mets	Radiological Response	Histopathological Response	Conversion Therapy	Survival Status
4	70’s	M	Transverse colon	Wild	V600E	LN	cPR	pCR	Yes	Alive with NED
6	40’s	F	Rectum	Wild	Wild	Peri	cPR	pCR	Yes	Alive with NED
9	50’s	M	Sigmoid colon	Wild	Wild	Peri	cPR	pCR	Yes	Alive with NED
11	60’s	M	Ascending colon	G13D	Wild	Peri	cPR	non-pCR	Yes	Alive with NED
13	70’s	F	Cecum	Wild	Wild	Liver	cPR	pCR	No	Alive with NED

*M*: male; *F*: female; *Mets*: metastases; *LN*: lymph node; *Peri*: peritoneum; *cPR*: clinical partial response; *pCR*: pathological complete response; *non-pCR*: non-pathological complete response; *NED*: no evidence of disease.

## 4. Discussion

In this study, we observed that 48% of patients with dMMR mCRC achieved an objective radiological response to pembrolizumab, with a significant proportion of those with cPR achieving pCR after metastasectomy. These findings are consistent with those of Ludford et al. [12], who demonstrated the potential dissociation between the radiological and pathological responses in ICI-treated mCRC patients. Although radiological imaging often suggested the persistence of disease, histopathological evaluation revealed no viable cancer cells in some cases, indicating that radiological assessments alone may not fully capture the treatment efficacy.

In patients with dMMR metastatic cancer, the response to pembrolizumab may persist even after treatment cessation. Le et al. reported that 7 out of 18 patients with dMMR metastatic cancer treated with pembrolizumab had radiologically detectable residual disease at the time of treatment cessation but remained progression-free at a median follow-up of 7.6 months [5]. This suggests that pembrolizumab not only increases the likelihood of conversion therapy but also offers a potential path to cure.

ICIs have been reported to demonstrate a higher pCR rate compared to that of chemotherapy in dMMR mCRC. Marolleau et al. analyzed 81 patients undergoing neoadjuvant treatment (12 treated with ICIs and 69 with chemotherapy) followed by metastasectomy. They reported pCR rates of 50.0% (6 out of 12 patients) for ICIs and 10.1% (7 out of 69 patients) for chemotherapy. Among eight patients who showed cPR after ICI treatment, 50.0% (four of eight patients) achieved pCR [25]. These findings highlight the high pCR rate associated with ICIs in dMMR mCRC. This also suggests that tumors that seem to persist on imaging may, upon histopathological evaluation, actually be completely absent.

ICIs activate the immune system to attack tumors [26,27,28,29,30]. ICIs can result in the destruction of tumor cells, leaving behind necrotic tissue, mucin, or inflammatory reactions. These residual components may appear as tumors on imaging studies, even though no viable cancer cells are present. Immune cell infiltration has been observed in analyses of resected melanoma and non-small-cell lung cancer after neoadjuvant ICI therapy [31,32]. Additionally, necrosis and/or acellular mucin has been noted in studies of resected dMMR CRC following ICI treatment [13,25]. This phenomenon, known as the discrepancy between radiological and histopathological assessments, underscores the limitations of imaging in accurately reflecting the pathological response to ICI therapy [12,13,14,15,16,25].

Our exploratory analysis of the five patients who underwent metastasectomy following cPR revealed potentially important associations between genetic status and pathological outcomes (Table 4). Notably, all four patients who achieved pCR had *KRAS* wild-type tumors, while the single patient with a residual viable tumor demonstrated a *KRAS* G13D mutation. Although this represents a small cohort that precludes formal statistical evaluation, this pattern raises the hypothesis that the *KRAS* mutational status may influence the concordance between the radiological and pathological responses in dMMR mCRC treated with pembrolizumab. Previous studies have suggested that *RAS* mutations may be associated with a reduced benefit from pembrolizumab in dMMR tumors [8], potentially explaining the persistent viable tumor despite the radiological complete response in our *KRAS*-mutated case. These preliminary observations highlight the need for larger, adequately powered studies to definitively establish whether genetic biomarkers can predict radiological–pathological discordance patterns and inform surgical decision making in this patient population.

The observed radiological–pathological discordance has significant clinical implications for treatment and surgical decision making. When imaging shows radiological partial response, clinicians must decide whether to continue pembrolizumab or proceed with resection. Our findings suggest that radiological partial response may underestimate the treatment efficacy, as patients with apparent residual disease on imaging may have actually achieved pCR. The 80% pCR rate among patients with cPR indicates that conventional imaging may overestimate the residual tumor burden. This discordance has important implications for clinical decision making; while current practice appropriately continues treatment and considers surgical resection in patients with radiological partial response, our findings suggest that some of these patients may have already achieved maximal therapeutic benefit. This raises questions about the optimal treatment duration and the timing of surgical intervention, as patients who have achieved pCR might benefit from earlier surgical evaluation rather than prolonged immunotherapy. The development of accurate biomarkers, such as circulating tumor DNA (ctDNA), to distinguish viable tumors from post-treatment inflammatory changes will be essential for optimizing the treatment duration and surgical timing decisions in patients with radiological partial response.

The discordance between the radiological and pathological responses observed in our study highlights the potential utility of circulating biomarkers for a more accurate assessment of the treatment response in immunotherapy. ctDNA has emerged as a promising liquid biopsy tool that may provide the real-time molecular assessment of the tumor burden and treatment response [33]. Unlike radiological imaging, which relies on morphological changes, ctDNA levels can reflect the actual burden of viable tumor cells, potentially offering the earlier and more sensitive detection of the treatment response [34,35]. In patients with dMMR tumors, monitoring the ctDNA clearance during pembrolizumab treatment could potentially identify patients achieving molecular complete response, despite radiological evidence of residual disease [36,37]. This approach may help clinicians distinguish between true residual disease and inflammatory changes or necrotic tissue that persist on imaging after effective immunotherapy. Future studies investigating the correlation between the ctDNA dynamics, radiological response, and pathological outcomes in pembrolizumab-treated dMMR mCRC patients could provide valuable insights for optimizing treatment monitoring and surgical decision making.

## 5. Limitations

There are several limitations in this study. First, the small sample size limits the generalizability of our findings, and the retrospective nature of this study precludes definitive conclusions regarding causality. Second, the short follow-up period may not have captured the long-term effects of pembrolizumab cessation. Third, the radiological assessment was performed by a single reviewer without independent dual review, potentially introducing inter-observer variability. Fourth, we applied conventional RECIST 1.1 criteria rather than immune-specific response criteria (iRECIST). iRECIST criteria, which account for delayed responses and pseudoprogression patterns characteristic of immunotherapy, might have provided a more accurate response assessment of the pembrolizumab-treated patients [17]. Fifth, the small cohort size of patients undergoing metastasectomy (*n* = 5) precluded formal statistical analyses, including multivariate analyses and adjustments for potential confounders. Such analyses would have been statistically underpowered and potentially misleading given the limited sample size. Therefore, our genetic correlation findings, particularly the observed association between the *KRAS* mutational status and pathological response, should be interpreted as exploratory observations that require validation in larger, adequately powered studies rather than definitive predictive relationships.

Despite these limitations, this study is one of the first to investigate the relationship between the radiological and pathological responses in dMMR mCRC patients treated with pembrolizumab. The novel finding that pCR can be achieved even in patients with cPR challenges the current reliance on imaging alone for evaluating treatment outcomes. This highlights the potential need for integrating additional assessment modalities, such as circulating biomarkers, into the treatment response assessment in these patients.

## 6. Conclusions

In conclusion, our findings demonstrate that the radiological partial response may underestimate the actual tumor response, as 80% of patients with cPR who underwent metastasectomy achieved pCR. This discordance between the radiological and pathological responses suggests that some patients with radiological partial response may have experienced the complete eradication of viable tumor cells. Further large-scale studies are warranted to validate these observations and investigate the utility of circulating biomarkers, such as ctDNA, for a more accurate assessment of the treatment response in immunotherapy settings.

## Figures and Tables

**Table 1:** Characteristics of 27 patients.

Characteristic		*n* (%)
Age, median (range), y		69 (37–81)
Sex	Male	14 (52)
	Female	13 (48)
Primary tumor location	Cecum	7 (26)
	Ascending	9 (33)
	Transverse	3 (11)
	Sigmoid	4 (15)
	Rectum	4 (15)
*KRAS* status	Wild type	23 (85)
	Mutant	4 (15)
*NRAS* status	Wild type	26 (96)
	Mutant	1 (4)
*BRAF* V600E	Wild type	17 (63)
	Mutant	9 (33)
	Unknown	1 (4)
Initial stage	II	4 (15)
	III	9 (33)
	IV	14 (52)
Type of metastases	Synchronous	14 (52)
	Metachronous	13 (48)
Number of metastatic organs	One	19 (70)
	Two or more	8 (30)
Lynch syndrome	Yes	3 (11)
	No	4 (15)
	Unknown	20 (74)
Timing of PEM	First line	12 (44)
	Second line and beyond	15 (56)
Metastasectomy after PEM	Absence	22 (81)
	Presence	5 (19)

*PEM*: pembrolizumab.

**Table 2:** Objective response and overall response.

Characteristic		*n* (%)
Objective response		13 (48)
Radiologic response	CR	3 (11)
	PR	10 (37)
	SD	9 (33)
	PD	5 (19)

*PEM*: pembrolizumab; *CR*: complete response; *PR*: partial response; *SD*: stable disease; *PD*: progressive disease.

## Data Availability

The datasets used and/or analyzed during the current study are available from the corresponding author upon reasonable request.

## Abbreviations

The following abbreviations are used in this manuscript:

dMMR	Mismatch repair deficient
mCRC	Metastatic colorectal cancer
ICI	Immune check point inhibitor
MSI	Microsatellite instability
MSS	Microsatellite stable
MSI-H	Microsatellite instability-high
pCR	Pathological complete response
PCR	Polymerase chain reaction
CT	Computed tomography
PFS	Progression-free survival
cCR	Clinical complete response
cPR	Clinical partial response
ctDNA	Circulating tumor DNA
